# Difference in cytokine production and cell cycle progression induced by Epstein-Barr virus Lmp1 deletion variants in Kmh2, a Hodgkin lymphoma cell line

**DOI:** 10.1186/1743-422X-11-94

**Published:** 2014-05-19

**Authors:** Charlotte Sueur, Julien Lupo, Philippe Mas, Patrice Morand, Véronique Boyer

**Affiliations:** 1 Université Grenoble Alpes, UVHCI, F-38000 Grenoble, France; 2 CNRS, UVHCI, F-38000 Grenoble, France; 3 Unit for Virus Host-Cell Interactions, Univ. Grenoble Alpes-EMBL-CNRS, 6 rue Jules Horowitz, Grenoble 38042, France; 4 CHU de Grenoble, CS 10217, 38043 Grenoble, Cedex 9, France; 5 EMBL Grenoble Outstation, F-38000 Grenoble, France; 6 UMR 912 SESSTIM, 23 rue S. Torrents, 13006 Marseille, France; 7 Hopitaux Universitaires de Strasbourg, Plateau Technique de Microbiologie, Laboratoire de Virologie, 1, place de l’hôpital, BP426 67091 Strasbourg, Cedex, France

**Keywords:** EBV, LMP1, Variant, Hodgkin’s Lymphoma, Cytokine, Cell cycle

## Abstract

**Background:**

Epstein-Barr virus (EBV) is associated with 20-40% of Hodgkin’s Lymphoma (HL) cases. EBV-encoded latent membrane protein 1 (LMP1) is a well-known oncogenic protein and two C-terminal deletion variants, del30-LMP1 and del69-LMP1, have been described in animal models to be more tumorigenic than the wild-type form. This work aims to detail the implication of LMP1 in the development of HL and to characterize the particular effects of these variants.

**Methods:**

We established HL-derived cell lines stably transfected with the pRT-LMP1 vector coding for the EBNA1 gene and allowing expression of the different LMP1 variants under the control of a doxycyclin-inducible promoter. Communication between cells was assessed by measuring the expression of various pro-inflammatory cytokines by flow cytometry after intracellular LMP1 and cytokine double staining. Proliferative properties of LMP1 variants were also compared by studying the repartition of cells in the different phases of the cell cycle after EdU incorporation combined to LMP1 and DAPI staining.

**Results:**

All LMP1 proteins induced the expression of several pro-inflammatory cytokines such as TNF-α, TNF-β, IL-6, RANTES/CCL5 and IFN-γ. However, the del30-LMP1 variant induced cytokine expression at a lower level than the other variants, especially IFN-γ, while the del69-LMP1 variant stimulated greater cytokine expression. In addition, we measured that all LMP1 proteins greatly impacted the cell cycle progression, triggering a reduction in the number of cells in S-phase and an accumulation of cells in the G2/M phase compared to the HL-non induced cells. Interestingly, the del30-LMP1 variant reduced the number of cells in S-phase in a significantly greater manner and also increased the number of cells in the G0/G1 phase of the cell cycle.

**Conclusion:**

Weak IFN-γ expression and specific alteration of the cell cycle might be a way for del30-LMP1 infected cells to escape the immune anti-viral response and to promote the development of cancer. The differences observed between the LMP1 variants reflect their own oncogenic properties and eventually impact the development of HL.

## Introduction

EBV is an ubiquitous tumor-causing virus infecting more than 90% of human adults worldwide with a life-long infection in B lymphocytes asymptomatically [1]. EBV infection in HRS cells presents a type II latency program, characterized by the expression of the latent membrane protein 1 (LMP1) and two other viral proteins, EBNA1 and LMP2 in addition to small RNAs, termed EBER 1 and 2 [2]. LMP1 is a multifunctional oncoprotein essential for EBV-induced B-cell proliferation and transformation *in vitro*[3], as it shares several features with CD40, a member of the tumor necrosis factor receptor family (TNFR). LMP1 activates the transcription factor nuclear factor-κB (NF-κB) by promoting turnover of IκBα, an important inhibitor of NF-κB, conferring the cells a protection against apoptosis. A direct link between LMP1 and cell cycle progression has also been shown in several studies although they were essentially focused on NPC cells or Burkitt lymphoma cell lines [4-7].

In the EBV-associated pathologies, different variants of LMP1 have been described. For example, a 30-bp deletion variant at the C terminal and corresponding to the CAO variant isolated from a NPC patient, was found to have increased potential to transform rodent fibroblasts and to induce tumors in nude mice when compared to wild-type LMP1 [8]. A 69-bp deletion LMP1 variant has also been described in NPC [9] and other lymphoproliferative disorders such as HL [10]. These deletions do not interfere with the stimulation of NF-κB [11]. However, the presence of such variants associated to the pathogenesis of several diseases leads to the hypothesis that polymorphisms within LMP1 gene might influence the susceptibility to develop EBV-associated tumors.

Hodgkin Lymphoma (HL) is a B cell lymphoma, characterized by a minority of neoplastic cells, the Hodgkin and Reed-Sternberg cells (HRS cells), and an extensive inflammatory background. Classical HRS cells derive from post-germinal center B cells, destined for apoptosis in the B cell selection process because of the lack of successful immunoglobulin gene rearrangement. In Western world, 20-40% of HL are associated with Epstein-Barr Virus (EBV) while in developing countries it reaches 70% [12]. Interestingly, EBV has been detected in 80-100% of HL arising in HIV patients, supporting the notion that this virus plays a pivotal role in the pathogenesis of this tumor [13]. Moreover, presence of the 30-bp deletion in LMP1 has been frequently associated with the HL disease in HIV infected individuals [13,14]. Recently, LMP1 has been shown to increase the DDR1 tyrosine kinase receptor in germinal center B cells, activating the expression of pro-inflammatory mediators and protecting lymphoma cells from cell death [15]. Besides, many studies have documented that HL is associated with disturbed cytokine production [16]. Cytokine and chemokine production may not only promote growth of HRS cells and help to evade immune surveillance, but they also cause the characteristic histology and the clinical symptoms of HL [17]. Cross-talk between HRS cells and surrounding lymphocytes has been studied for many years, and this interaction appeared to play an important role for the pathogenesis of HL [18]. Few studies have documented the impact of EBV infection on HL development. HL frozen tissues [19] or derived cell lines infected by EBV *in vitro* or transiently transfected by a constitutive expressed LMP1 vector were used [20-24]. However, results obtained from these studies were difficult to interpret since either there were not quantitative or the cell lines did not express LMP1 until a membrane signal was applied (CD40 ligand and IL4), leading to morphological studies where LMP1 was linked to the formation of multinuclear cells or showing differentially expressed proteins by microarray RNA assays, not confirmed by protein expression techniques. Other studies about LMP1 genetic diversity from samples derived from HL patients focusing mainly on LMP1 variant origin and activation of the NF-κB pathway were also conducted [25-27]. However, the impact of the LMP1 polymorphism on the HL cells has not been documented.

In this study, we investigated whether WT-LMP1 and the deletion variants del30-LMP1 and del69-LMP1 could modulate cytokine expression and cell cycle progression in KMH2 – a HL derived cell line – to analyze the impact of LMP1 polymorphism on the development of HL.

## Results

### Characterization of the KMH2-pRT-LMP1 established cell lines

**Figure 1:**
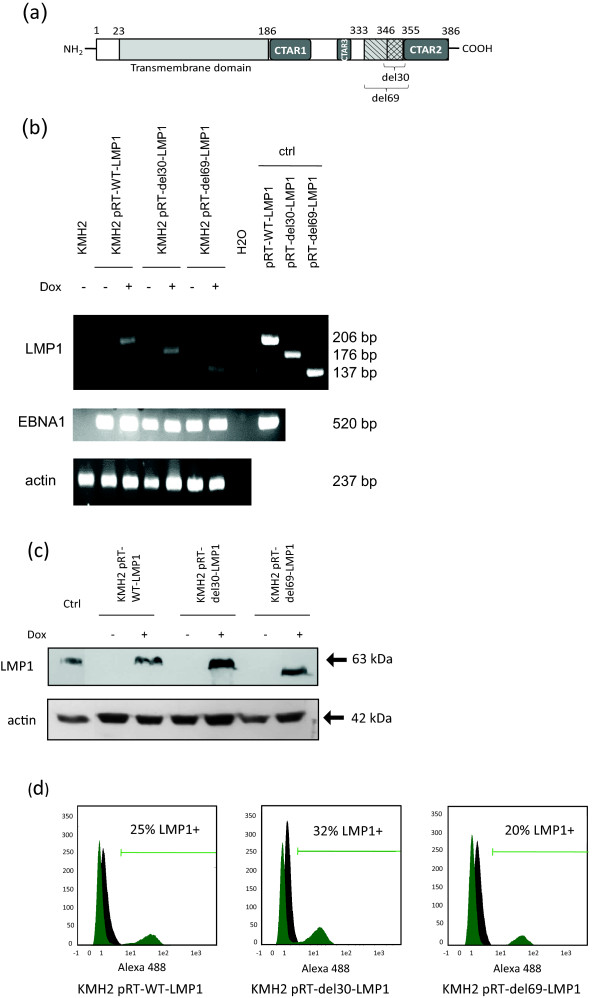
**Characterization of the KMH2-pRT-LMP1 established cell lines. (a)** Linear representation of WT-LMP1 and the 10 and 23 amino-acid deletions corresponding to the del30 and del69-LMP1 variants. **(b)** RT-PCR or **(c)** Western blot of EBNA1 and LMP1 in non-induced cells and in doxycyclin-induced cells expressing WT-LMP1, del30-LMP1 and del69-LMP1 variants. 293-HEK cells transiently transfected with pcDNA-WT-LMP1 were used as positive control for immunoblotting. **(d)** Comparison of LMP1 expression by flow-cytometry in the KMH2-pRT-LMP1 cells. Doxycyclin-induced cells are represented in green and non-induced cells in black. Data are representative of 3 experiments.

In order to study the impact of different LMP1 deletion variants on the behavior of the KMH2 HL cell line, we established three cell lines stably transfected with the pRT-LMP1 vector coding for either the wild-type form of LMP1 (WT-LMP1) or deleted variants (del30-LMP1; del69-LMP1) (Figure 1a). After electroporation and three weeks of hygromycin selection, presence of the plasmid and expression of viral genes were assessed by inducing cells with doxycyclin for 24 h. Expectedly, RT-PCR showed that the EBNA1 gene was constitutively expressed in the three KMH2-pRT-LMP1 cell lines but not in the KMH2 cells. LMP1 was only expressed in presence of doxycyclin, as shown by RT-PCR (Figure 1b). A shift can be observed between the three PCR products of the LMP1 amplification corresponding to the 30-bp and 69-bp deletions in the LMP1 gene. LMP1 inducible-expression was also observed by western-blotting (Figure 1c) showing no significant difference in LMP1 expression normalized to actin (actin/LMP1 ratio: WT-LMP1 ×1.89; del30-LMP1 × 1.54; del69-LMP1 × 1.75). The precise number of cells expressing LMP1 in the three cell lines was determined by flow-cytometry (Figure 1d). On average, 25% of the KMH2-pRT-WT-LMP1 cells, 32% of the KMH2-pRT-del30-LMP1 cells and 20% of the KMH2-pRT-del69-LMP1 expressed LMP1 compared to non-induced cells. These low rates of cells expressing LMP1 could be due to heterogeneity in the LMP1 expression level or to the presence of hygromycin resistant KMH2 cells. Attempts to enrich or clone LMP1 expressing cells (by selecting NGFR-expressing cells or subcloning) were unsuccessful since KMH2 cells express low level of endogenous NGFR and did not grow at low density. In order to study the impact of LMP1 variants on HL cells, we used flow cytometry to gate selectively the LMP1 positive cells among the established cell lines in all the next experiments.

### LMP1 induces expression of several cytokines in KMH2 cells

Communication between HRS cells and the surrounding immune cells plays a major role in the development of HL. To examine the influence of LMP1 on HRS cytokine secretion, we studied intracellular cytokine expression by flow cytometry in KMH2 cells expressing LMP1 or stimulated by phorbol-myristate-acetate and ionomycin (PMA-iono) as positive control. We choose to study different cytokines involved in inflammatory processes, recruitment of immune cells and HL pathogenesis. After 24 h of doxycyclin induction, all KMH2-pRT-LMP1 cells expressed TNF-α, TNF-β, IL-6, RANTES/CCL5 and IFN-γ (Table 1). On the contrary, none of the LMP1 variants induced expression of TGF-β, IL-8, IL-9, IL-1α and IL-1RA, while stimulation of KMH2 with PMA-ionomycin induced all these cytokines (Table 1). Of note that production of cytokines was not modified by addition of doxycyclin in the non-transfected KMH2 (data not shown). Thus, LMP1 induced the expression of several cytokines in HL cells, promoting the establishment of an inflammatory microenvironment in favor of the tumor development.

**Table 1:** Cytokine expression induced in Hodgkin’s lymphoma-derived cells

	**KMH2 PMA-iono**	**KMH2 pRT-WT-LMP1**	**KMH2pRT-del30-LMP1**	**KMH2 pRT-del69-LMP1**
TNF-α	+	+	+	+
TNF-β	+	+	+	+
IL-6	+	+	+	+
RANTES/CCL5	+	+	+	+
IFN-γ	+	+	+	+
TGF-β	+	-	-	-
IL-8	+	-	-	-
IL-9	+	-	-	-
IL-1α	+	-	-	-
IL-1RA	+	-	-	-

Cytokine expression was assessed by flow cytometry analysis after induction of KMH2 cells with PMA-ionomycin or doxycyclin for 24 h.

### del30-LMP1 induces cytokine expression at a lower level than the other LMP1 variants

**Figure 2:**
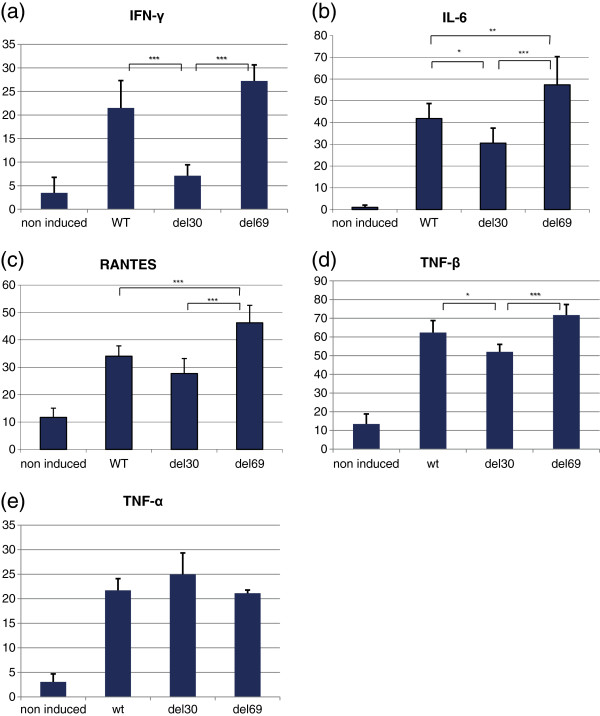
**Cytokine expression is differently regulated by LMP1 variants.** Cytokines expressed by KMH2-pRT-LMP1 cells (non-induced, WT, del30 and del69) were intracellularly stained and detected by flow-cytometry 24 h after induction of LMP1 expression. The percentages of cells expressing **(a)** IFN-γ, **(b)** IL-6, **(c)** RANTES/CCL5, **(d)** TNF-β and **(e)** TNF-α were compared. All cytokines were significantly more expressed in LMP1 expressing cells compared to non-induced cells with an ANOVA analysis (of note that expression of IFN-γ was significantly higher in KMH2-pRT-del30-LMP1 cells compared to the control unstimulated KMH2 cells with an ANOVA analysis followed by a Dunnett post-test). Supplemental data showing LMP1 and cytokine labeling dot plots are provided as Additional file 1.

Furthermore, we investigated the effect of the different LMP1 deletion variants on HRS cytokine secretion. LMP1 and cytokine labeling dot plots are shown in Additional file 1. IFN-γ was significantly induced by WT-LMP1 (21.5 ± 5.8%) and del69-LMP1 (27.2 ± 3.4%) compared to LMP1 non-expressing cells (3.5 ± 3.3%) (Figure 2a). Interestingly, del30-LMP1 increased the number of IFN-γ producing cells to a significantly lesser extent (7.1 ± 3.4%) compared to the WT-LMP1 and del69-LMP1 forms, although to a significant amount compared to the non-induced cells. Likewise, the three LMP1 variants were able to induce IL-6 expression compared to non-induced cells (1.1 ± 1.0%; Figure 2b). However, the number of del30-LMP1 cells was significantly lower (30.5 ± 6.9%) than the two other variants WT-LMP1 (41.9 ± 6.9%) and del69-LMP1 (57.4 ± 12.9%). In addition, a significant greater number of del69-LMP1 cells expressed IL-6 compared with the other variants (Figure 2b). The same observation can be made with RANTES/CCL5, whose expression is induced by all LMP1 variants compared to non-induced cells, and in a significantly higher proportion with the del69-LMP1 variant (control cells 11.7 ± 3.4%, WT-LMP1 34.0 ± 3.8%, del30-LMP1 27.7 ± 5.5%, del69-LMP1 46.2 ± 6.3%) (Figure 2c). Two members of the TNF family were then analyzed. TNF-β expression was induced by all LMP1 variants compared to KMH2 control cells (13.4 ± 5.4%) with a significantly smaller percentage of del30-LMP1 cells expressing TNF-β (52.0 ± 4.0%) in relation to the two other variants WT-LMP1 (62.3 ± 6.4%) and del69-LMP1 (71.7 ± 5.6%) (Figure 2d). TNF-α expression was induced by LMP1 compared to non-induced KMH2 cells (3.0 ± 1.6%; Figure 2e). No significant difference could be observed between the LMP1 variants (WT-LMP1 21,7 ± 2.4%; del30-LMP1 24,9 ± 4.4%; del69-LMP1 21.1 ± 0.6%).

To sum up, del30-LMP1 tended to induce cytokine expression at a lower level compared with both forms: WT-LMP1 and del69-LMP1. Nonetheless, del69-LMP1 seemed to induce more cytokine expression than the two other LMP1 variants.

### LMP1 variants influence differently cell cycle progression

**Figure 3:**
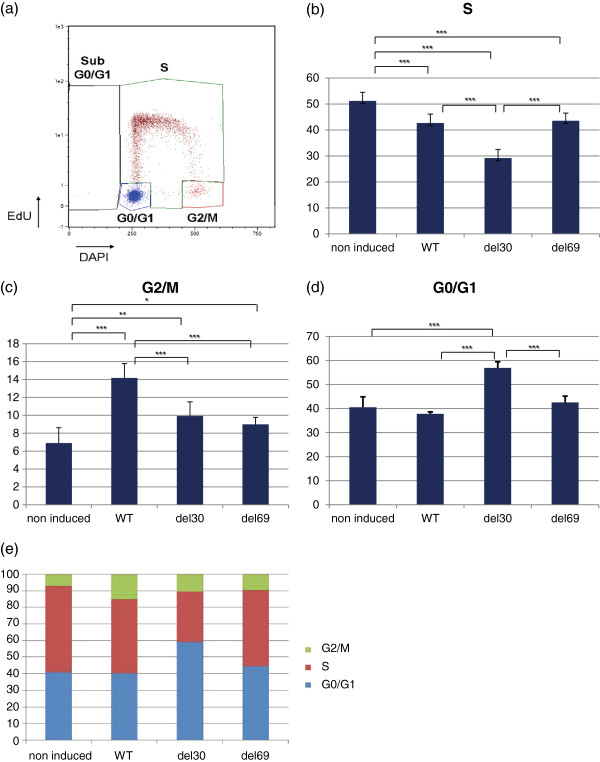
**LMP1 influences cell cycle progression in the KMH2 Hodgkin’s lymphoma cell lines.** Percentage of cells in the different phases of cell cycle was assessed by measurement of EdU incorporation and DAPI staining by flow cytometry after induction of LMP1 expression in the KMH2-pRT-LMP1 cell lines (non-induced, WT, del30 and del69). **(a)** Example of cell cycle dot plot, **(b)** Cells in S phase, **(c)** Cells in G0/G1 phase and **(d)** Cells in G2/M phase. **(e)** Repartition of cells in the different phases of the cell cycle represented in a stacked histogram.

The LMP1 deletion variants have been shown to be more tumorigenic than the WT-LMP1 [8,28]. In order to study the effect of LMP1 variants on the cell cycle progression in the KMH2 cell line, percentages of cells in the different phases of cell cycle were determined by flow cytometry, using DAPI and EdU incorporation as shown in Figure 3a. Twenty four hours after induction, all LMP1-expressing cells show a significantly smaller percentage of cells in S phase than the control cells (Figure 3b). We verified that the observed effects were actually due to the expression of LMP1 rather than the presence of doxycyclin in the medium by inducing non-transfected KMH2 cells with doxycyclin for 24 h. Indeed, no difference could be detected between doxycyclin-induced and non-induced KMH2 cells (data not shown). It is worth noting that the expression of del30-LMP1 variant reduced the percentage of cells in S phase more significantly than the two other LMP1 variants (Figure 3b). If all LMP1 variants had an enhanced percentage of cells in G2/M phase compared to the control cells (6.9 ± 1.7%), WT-LMP1 induced a significantly higher accumulation of cells in G2/M phase (14.2 ± 1.6%) than the deletion variants del30-LMP1 (9.9 ± 1.6%) and del69-LMP1 (9.0 ± 0.8%) (Figure 3c). Interestingly, expression of del30-LMP1 promoted a significant accumulation of cells in G0/G1 phase (56.9 ± 2.6%) compared to the control cells (40.5 ± 4.4%) while WT-LMP1 (37.8 ± 0,8%) and del69-LMP1 (42.6 ± 2.6%) did not differ from non-induced cells (Figure 3d). Percentage of sub-G0/G1 cells was slightly increased by the expression of WT-LMP1 (3.16 ± 1.76%) or del69-LMP1 (2.85 ± 0.99%) protein compared to control cells (1.14 ± 0.67%)(data not shown). However, number of del30-LMP1 cells in sub-G0/G1 phase was not significantly different compared with control cells (2.33 ± 0.54%).

Thus, all variants reduced the S phase and accumulated cells in the G2/M phase, suggesting a blockade at the G1/S checkpoint and a block to completion of mitosis in the KMH2 cells (Figure 3e). The del30-LMP1 variant significantly reduced the number of S-phase cells and further increased the G0/G1-phase cells.

## Discussion

In the context of HL, where tumor cells represent only 0.1 to 1% of the cells surrounded by fibroblasts and immune cells, communication between cells mediated by cytokines and chemokines is of upmost importance. LMP1 is a well-known oncogene involved in many cellular signaling pathways and thus able to interfere in cytokine secretion. Previously, LMP1 has been shown to induce IL-6 and IL-8 secretion in epithelial cells [29] via the NF-κB pathway [30] and IL-10 in Burkitt lymphoma cells [31]. In LCL, LMP1 triggers cell proliferation by inducing cytokines such as CCL3 and CCL4 through the JUN-kinase pathway [32], and also uses the NF-κB pathway to stimulate IFN secretion [33]. In this study, we showed that LMP1 induced significantly the expression of several cytokines in LMP1 positive KMH2 HL-derived cells, such as IFN-γ, IL-6, RANTES/CCL5, TNF-β and TNF-α. These cytokines are mainly pro-inflammatory, attracting immune cells in the microenvironment and known to be implicated in the development of HL [17,34]. Vockerodt et al. [24] showed that LMP1 induced high levels of IL1, IL8 and RANTES mRNAs in primary human tonsillar germinal center B cells (CD10+ cells). We did not find IL8 and IL1 elevated amounts in the LMP1 positive KMH2 cell lines. Whether this discrepancy is due to the different techniques used or to the cells still remains to be determined.

Little is known about the influence of LMP1 on cytokine production in the particular context of HL. An histological study showed that IL-6 was more often expressed in LMP1-positive HRS cells compared to the negative ones [35]. Showing differences in cytokine expression between the three LMP1 positive forms could be of interest to HL. While a significantly higher number of del69-LMP1 cells expressed IL-6 and RANTES, del30-LMP1 cells expressed IFN-γ, IL-6 and TNF-β in a significantly lesser extent than the other variants. IFN-γ is involved in the anti-viral response against EBV-infected cells. Weak IFN-γ expression might be a way for del30-LMP1 infected cells to escape the immune anti-viral response. Besides, on the one hand, the presence of cytokines is necessary for cell survival and tumor promotion. Yet, on the other hand, an excessive cytokine secretion stimulates the recognition and degradation of EBV-infected cells by the immune system. The low level of cytokines expressed in del30-LMP1 KMH2 cells could be a mechanism for EBV to promote the development of HL while escaping the immune surveillance. These observations are consistent with the high frequency of del30-LMP1 variant observed among HIV positive people developing a HL [13,14]. On the contrary, the high proportion of del69-LMP1 HL cells to express pro-inflammatory cytokines could trigger their recognition by the immune system and would explain the low frequency of the del69-LMP1 variant *in vivo*, described only sporadically in NPC or some lymphoproliferative diseases. Prognostic significance of pretreatment serum cytokines in HL has been described [36]. In particular, they show that serum levels of IL6 and IL2R may be used to identify patients with HL at risk for early-disease relapse. Since we found that IL6 was less induced by the del30-LMP1 variant, it thus would be of great interest to determine *in vivo*, if the IL6 level in serum may be linked to the presence of del30-LMP1 variant and of early-disease relapse.

Oncogenic LMP1 has also a great influence on cell proliferation and survival by contributing to cell cycle progression. Here we show for the first time in HL cells, that all LMP1 variants interfered with cell cycle progression. LMP1 caused a decrease in the S-phase cell population, particularly significant with the del30-LMP1 variant and an increase of the G2/M-phase cells. The del30-LMP1 variant provoked an additional accumulation of cells in the G0/G1 phase. These observations were consistent with previous studies performed in NPC cell lines and LCLs with WT-LMP1 or a NPC-derived variant, carrying the 30 bp deletion [4,7]. LMP1 has been described to impair G2 checkpoint leading to a possible accumulation of cells with unrepaired DNA damages [4] or to promote cell cycle progression by accelerating the G1/S transition [5,37]. On the other hand, LMP1 has also been described to have cytostatic or even cytotoxic effects when expressed at high levels [7,38]. A recent work suggests that the ability of LMP1 to exert both cytotoxic and pro-proliferative properties is necessary during the transformation process [39]. While LMP1 induces cell death in the microenvironment, only EBV infected cells expressing the oncogenic LMP1 survive [39]. The fact that LMP1 variants do not elicit the same effect on cell cycle progression suggests that they have different impact on cell cycle checkpoints. This could directly reflect on their oncogenic potential. Besides, cytokines themselves can influence the cell cycle progression. For example, IL-8 is often overexpressed in cancers and contributes to the tumor development by promoting the G1/S transition of cell cycle [40]. It would be interesting to further investigate the cellular mechanism involved in cell cycle progression with each LMP1 variant and particularly the impact of the LMP1-induced cytokines.

In conclusion, this study brings new insights into the impact of LMP1 on cytokine expression and cell cycle progression in HL. We highlight differences between LMP1 variants which could partly be responsible for their respective oncogenic properties and explain their implication as risk factors in the development of HL.

## Materials and methods

### Plasmid constructs

We performed an overlapping PCR using the p2167 plasmid (kindly provided by W. Hammerschidt, Munich, Germany), in order to create the 30-bp and 69-bp deletions in the BNLF1 gene coding for LMP1 [41]. The p2167 plasmid contains 9.5 kb of the EBV genome, as described [42]. Two DNA fragments surrounding the deletions (respectively in position 1190-1229 and 1161-1229) were amplified by PCR with a high fidelity polymerase (Pfu, Promega, Fitschburg, WI, USA). The following primers were used,

P1: 5′-CGTGCTGCTAGCTCTTAGTTTCTGGGTGTG-3′;

P2a: 5′-GCCCGCCTTTGATGACAGACGGAGGCGGCGGTGATCCACACCTTC-3′;

P2b: 5′-CAATTGACGGAAGAGGTTGAAAACAAAGGAGGTGATCCACACCTTCCTACGC-3′;

P3a: 5′-TCCGTCTGTCATCAAAGGCGGGC-3′;

P3b: 5′-TCCTTTGTTTTCAACCTCTTCCGTCAATTG-3′;

P4: 5′-GCCCTTTGTATACTCCTACTGATGAGTAAGTATTACACCC-3′.

First, two PCRs were performed with 50 ng of the p2167 plasmid in a total volume of 50 μl, with primers P1 + P2a and P3a + P4 for the 30-bp deletion or P1 + P2b and P3b + P4 for the 69-bp deletion. After 5 min of denaturation at 95°C, 10 cycles of 30 sec at 95°C, 30 sec at 55°C and 2 min at 72°C were carried out, followed by an elongation step for 5 min at 72°C. PCR products were purified by QIAEX purification kit (Qiagen, Hilden, Germany), quantified by the nanodrop system (Labtech, Uckfield, United-Kingdom) and equimolarly mixed to be submitted to a second PCR. After 10 cycles with the same program but without primers, allowing the first two PCR products to hybridize, external primers P1 and P4 were added for another 20 cycles. The final 1.6 kb PCR product containing the 30-bp or 69-bp deletion was reintroduced into the original p2167 plasmid in the NheI and Bst1107I restriction sites and subcloned into the Sfi-I sites of the pRT-LMP1 vector (kindly provided by J. Feuillard, Limoges, France) with the In-Fusion HD cloning kit (Clontech, Mountain View, CA, USA). This vector includes the EBNA1 and hygromycin B resistance genes, that favour maintenance of the vector as an episome in the cells, and allow selection of transfected cells respectively. A bidirectional tetracyclin-inducible promoter drives the expression of LMP1 and NGFRt [43]. The pcDNA-WT-LMP1 plasmid is a kind gift from F. Grässer (Hambourg, Germany).

### Cell culture, transfection and induction

293-HEK cells (human embryonic kidney cell line) were cultured in DMEM supplemented with heat inactivated fetal bovine serum 10% and antibiotics (100 units/ml penicillin/streptomycin and 1 μg/ml ofloxacin). KMH2 cells (EBV-negative Hodgkin Lymphoma-derived cell line obtained from DSMZ, Braunschweig, Germany) were cultured in RPMI supplemented with 10% heat inactivated fetal bovine serum and antibiotics (100 units/ml penicillin/streptomycin, 1 μg/ml ofloxacin), at 37°C in a 5% CO_2_ atmosphere. For transient transfection of pcDNA-WT-LMP1 in 293-HEK cells, 5.10^5^ cells were plated in a 35-mm petri dish and transfected with Lipofectamine 2000 (Invitrogen, Carlsbad, CA, USA) in the ratio of 10 μl transfection reagent for 4 μg of DNA, following the manufacturer’s instructions. Establishment of KMH2 cell lines stably transfected with the three pRT-LMP1 plasmids (pRT-WT-LMP1, pRT-del30-LMP1 and pRT-del69-LMP1) was performed with the Amaxa electroporation system (Amaxa, Cologne, Germany), according to the manufacturer’s instructions. Five millions KMH2 cells were electroporated with 5 μg of DNA, using the Nucleofactor kit-T (Amaxa) and the T-001 program. Cells were then resuspended in 5 ml of fresh RPMI with 20% FBS, supplemented with 1X non-essential amino-acids, 10 mM Hepes and 1 mM sodium pyruvate. Hygromycin was introduced in the medium 48 h after the transfection at 200 μg/ml. After three weeks of selection, LMP1 expression was assessed by flow cytometry after inducing the cells with 1 μg/ml doxycyclin (Clontech) for 24 h, as described elsewhere [43].

### Western-blotting

Five millions KMH2-pRT-LMP1 cells were grown in presence or not of doxycyclin for 24 h. Cells were lysed in a buffer composed of 50 mM Tris pH 6.8, 2% SDS, 10% glycerol and protease inhibitors. Lysates were sonicated and total proteins were quantified with BCA kit (Pierce, Rockford, IL, USA) according to the manufacturer’s instructions. Fifty micrograms of total proteins were separated on a 9% SDS-PAGE gel, transferred on a PVDF membrane and revealed by western-blot analysis with an anti-LMP1 antibody (monoclonal CS.1-4, Dako, Glostrup, Denmark, 1:100) and a rabbit polyclonal anti-actin (Sigma, St Louis, MO, USA; 1:400). Secondary HRP-conjugated anti-mouse (1:20000), anti-rabbit antibodies (1:10000) were purchased from Invitrogen). The signal was developed by Supersignal West Pico Chemiluminescent Substrate (Pierce). The intensity of protein bands was quantified with Adobe Photophop CS3 software.

### RNA extraction and RT-PCR

Total RNA was extracted from 5.10^6^ KMH2-pRT-LMP1 cells with TRI-Reagent (Euromedex, Souffelweyersheim, France), quantified with a nanodrop ND1000 instrument system (Labtech) and treated with Turbo-DNase (Ambion, Austin, TX, USA). cDNA was synthesized from 1 μg total RNA with random hexamers, using the superscript III (Invitrogen) in a total volume of 20 μl. PCRs were then performed on 1 μl of cDNA, with 0.25U of the Phusion polymerase (Thermo-Scientific, Waltham, MA, USA) in the provided buffer, in presence of 200 μM dNTP and 200 μM of specific primers. Primer sequences are: LMP1-forward, 5′-CCTCATAGCCCTAGCGACTC-3′ ; LMP1-reverse, 5′-GTCGTCATCATCTCCACCGG-3′ ; EBNA1-forward, 5′-CCGCAGATGACCCAGGAGAA-3′ ; EBNA1-reverse, 5′-TGGAAACCAGGGAGGCAAAT-3′ ; β-actin-forward, 5′-CGTGATGGTGGGCATGGG-3′ β-actin-reverse, 5′-CTGGGTCATCTTCTCGCG-3′. After a denaturation step of 3 min at 94°C, the following cycle was applied to the samples : 30 sec at 94°C, 30 sec at 60°C (for EBNA1, T_H_ was 56°C), 30 sec at 72°C, repeated 26 times for the actin amplification and 30 times for LMP1 and EBNA1 amplifications. A final elongation step was performed for 5 min at 72°C. PCR products were migrated on a 2% agarose gel in 1X TAE buffer and visualized with ethidium bromide staining (Invitrogen).

### LMP1 staining and intracellular cytokine detection

KMH2 cells were either treated by doxycyclin (1 μg/ml) to induce LMP1 expression or stimulated with 50 ng/ml PMA (Sigma) and 1 μg/ml ionomycin (Sigma) to be used as positive control for cytokine expression during 24 h. Throughout the last 4 h of doxycyclin or PMA-ionomycin induction, cells were treated with 10 μg/ml brefeldin A (BioLegend, San Diego, CA, USA) and 2 μM monensin (Sigma) to block secretion of cytokines. Cells were collected and fixed with fixation buffer (BioLegend) for 20 min at room temperature. Cells were then permeabilized by two centrifugations (for 8 min at 350 g) with permeabilization wash buffer (BioLegend) allowing sequential double immunolabeling of LMP1 and cytokines of interest. LMP1 staining was performed primarily as follows: cells were saturated for 30 min in 100 μl RPMI containing 10% human plasma (kindly provided by EFS Rhône-Alpes, Grenoble, France) and 0.1% triton X-100, incubated for 30 min with the anti-LMP1 antibody (1:100) and washed twice with 5 ml of RPMI. Cells were then incubated for 30 min with an Alexa A488 or A594-conjugated anti-mouse antibody (Invitrogen, 1:2500) and washed twice with 5 ml of RPMI. An anti-mouse secondary antibody free Fab sites was finally satured with a mouse hybridoma supernatant (clone D3210, kindly provided by E. Drouet, UVHCI, Grenoble, France). In a second step, intracellular cytokine staining was carried out by resuspending cells in 100 μl of permeabilization wash buffer supplemented with the anti-cytokine antibody and incubated for 20 min at room temperature, protected from light. Cells were then washed twice with 2 ml of permeabilization wash buffer and finally resuspended in cell staining buffer (BioLegend) for flow cytometry analysis on a MACSQuant VYB (Miltenyi Biotech GmbH, Bergisch Gladbach, Germany). Anti-cytokine antibodies used were APC-conjugated anti-human TNF-α (clone Mab11) or its mouse isotype control (clone MOPC21), PE-conjugated anti-human IL-6 (clone MQ2-13A5), anti-human IL-8 (E8N1), anti-human TNF-β (clone 359-81-11), anti-human TGF-β (TW4-2 F8), anti-human IL-1α (364-3B3-14), rat isotype control (clone RTK2071) and mouse isotype control (clone MOPC21) from BioLegend, PE-conjugated anti-human IFN-γ (clone 4S.B3), anti-human IL-9 (MH9A3) and anti-human RANTES/CCL5 (clone 2D5) from BD Pharmingen (San Diego, CA, USA), or FITC-conjugated anti-human IL1-RA (CRM17) and its mouse isotype control (P3.6.2.8.1) from eBioscience (San Diego, CA, USA).

### Cell cycle and proliferation

In order to determine the percentage of cells in the different phases of cell cycle, we used the EdU-click 555 (Jena Bioscience, Jena, Germany) combined with LMP1 and DAPI staining. EdU is a nucleoside analogue to thymidine incorporated into DNA, it reveals the proliferating cells in S phase. DAPI measures the total quantity of DNA in the cells and the LMP1 labelling enables to select only the LMP1-expressing cells for our study. Cells were induced with doxycyclin for 24 h and, during the last 150 min, 10 μM EdU was added in the culture medium. Cells were, then, fixed with fixation buffer and permeabilized with permeabilization wash buffer (BioLegend). For EdU revelation, cells were resuspended in the cocktail-click containing TAMRA-azide, incubated for 30 min at room temperature protected from light and washed twice with 6 ml PBS. LMP1 staining was performed as described previously for intracellular cytokine detection and cells were finally incubated in PBS with 1 g/L glucose, DAPI 1:1000 and 100 μg/ml RNAseA until flow cytometry analysis.

### Statistical analysis

Means, standard deviations and p-values were calculated with the GraphPad InStat3 software (San Diego, CA, USA). All bar graphs represent means *as per* at least three independent experiments for cytokine detection and at least five independent experiments for cell cycle studies. Error bars represent standard deviations. To assess statistical differences, means were compared through a one-way ANOVA test followed by a Tukey post-test for multiple comparison significance test between the analyzed groups. When specified, for single comparison of a population with the control cell group, the ANOVA test was followed by a Dunnett post-test. * stands for p < 0.05, ** for p < 0.01 and *** for p < 0.001.

## Competing interests

The authors declare that they have no competing interests.

## Authors’ contribution

CS, JL and VB designed the experiments and analyzed the data; CS, JL, and PhMa performed the experiments; PM provided critical expertise in the EBV field; CS and VB wrote the manuscript; and all the authors approved the manuscript.

## Supplementary Material

Additional file 1 **Cytokine expression by KMH2-pRT-LMP1 expressing cells.** Cytometric data plots are presented for each cytokine expressed by the three KMH2-pRT-LMP1 cell lines, with or without induction of LMP1 expression, compared to an isotypic control antibody. X-axis shows LMP1 expression while Y-axis represents cytokine expression. Percentages of cells in each quadrant are given in black. LMP1-positive cells (right upper and lower quadrants) are selectively gated and considered 100% in order to calculate the percentage of LMP1-positive cells expressing the cytokine of interest, indicated in red. Experiments have been conducted in triplicates and pictures shown here represent only one out of three experiments. Click here for file
