# Supplementary material for: Difference in cytokine production and cell cycle progression induced by Epstein-Barr virus Lmp1 deletion variants in Kmh2, a Hodgkin lymphoma cell line
Source: Virol J. 2014 May 19;11:94. doi: 10.1186/1743-422X-11-94 (PMC4035821; doi:10.1186/1743-422X-11-94)

TNF-beta

KMH2-WT-LMP1

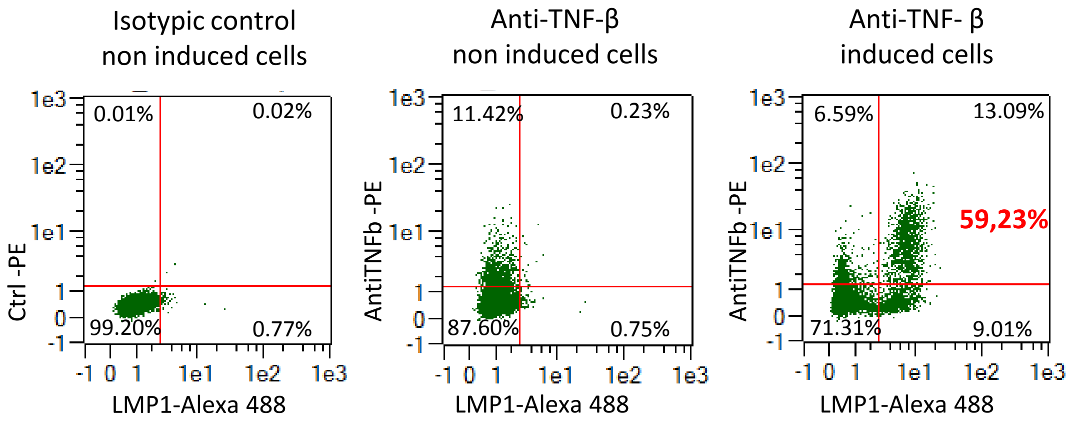

KMH2-del30-LMP1

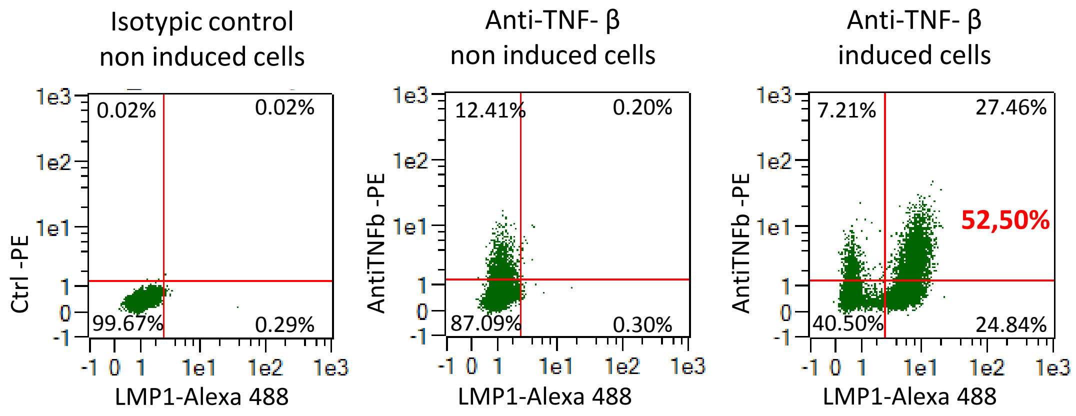

KMH2-del69-LMP1

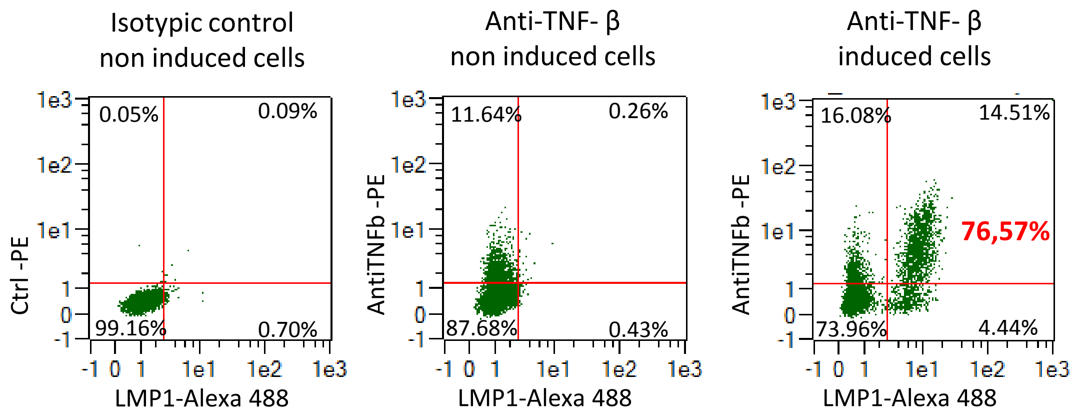

TNF-alpha

KMH2-WT-LMP1

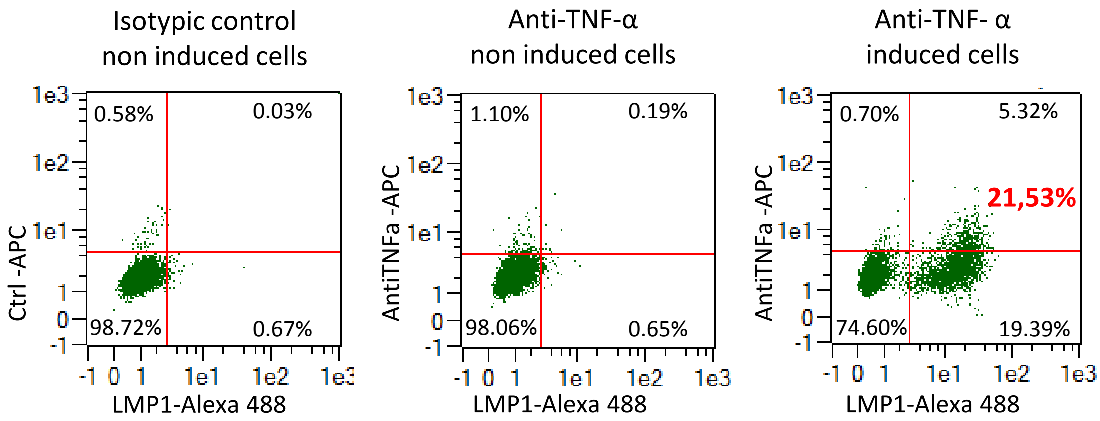

KMH2-del30-LMP1

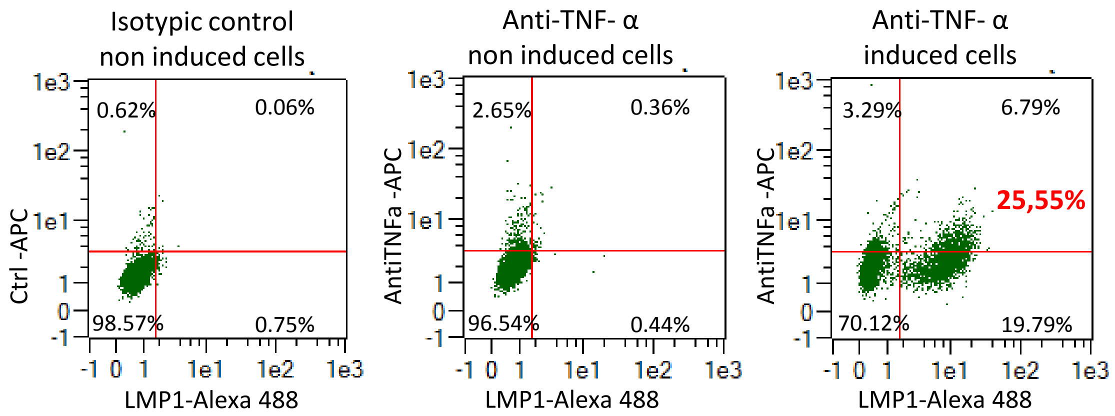

KMH2-del69-LMP1

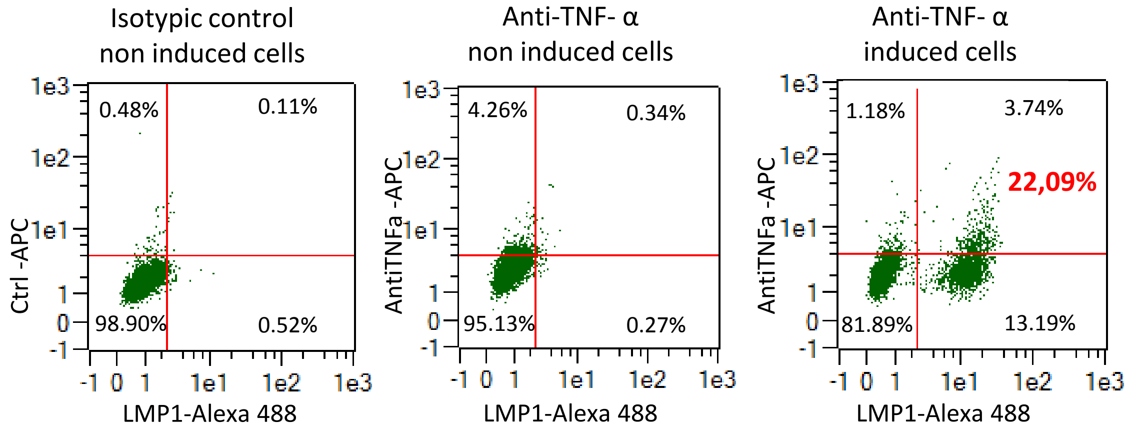

IFN-gamma

KMH2-WT-LMP1

Isotypic control non induced cells | Anti-IFN-g non induced cells | Anti-IFN-g induced cells

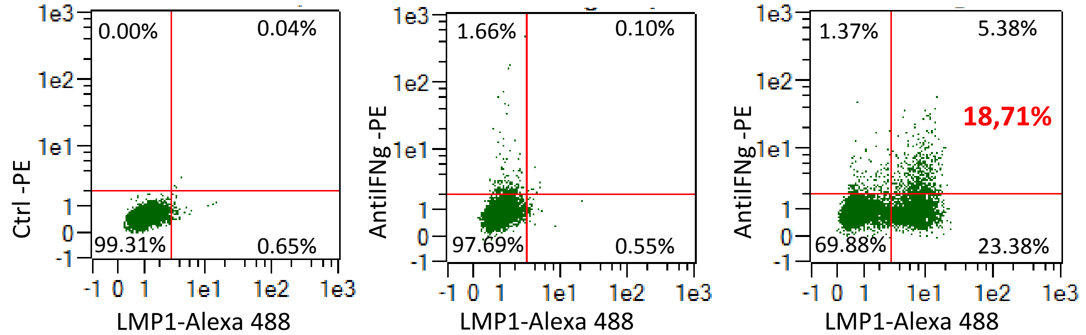

KMH2-del30-LMP1

Isotypic control non induced cells | Anti-IFN-g non induced cells | Anti-IFN-g induced cells

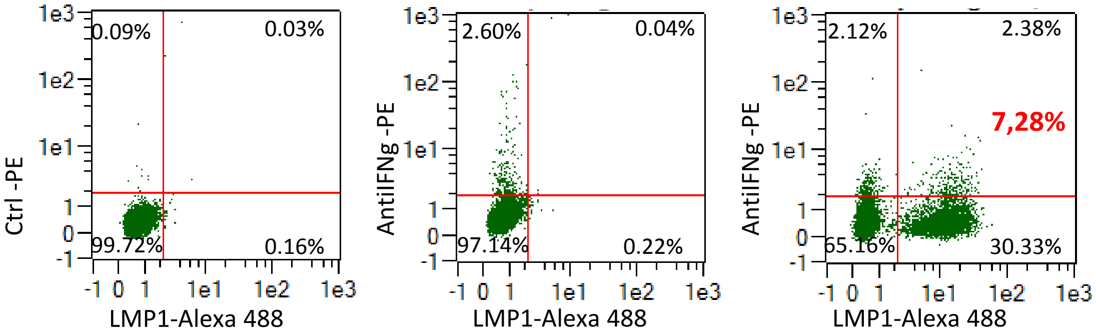

KMH2-del69-LMP1

Isotypic control non induced cells | Anti-IFN-g non induced cells | Anti-IFN-g induced cells

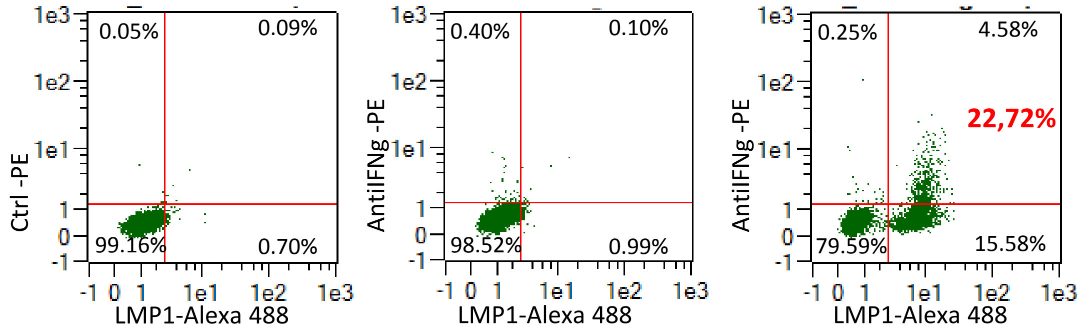

IL-6

KMH2-WT-LMP1

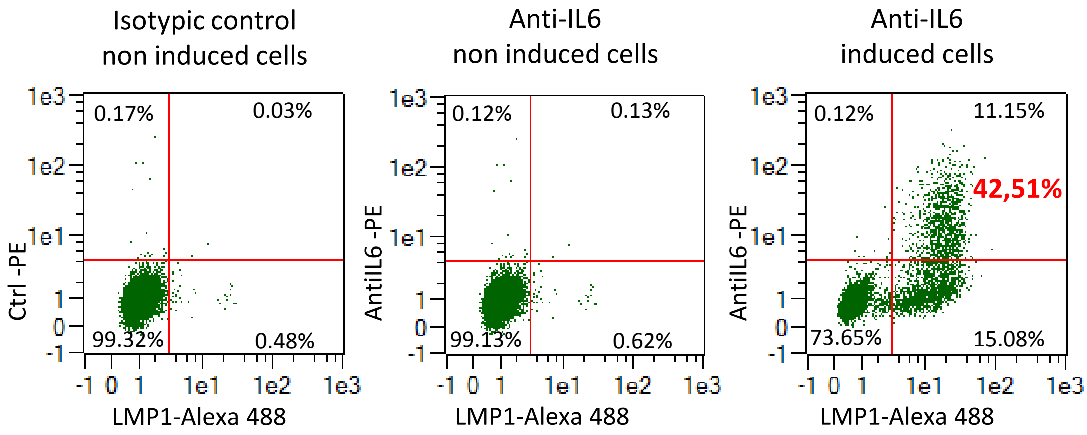

KMH2-del30-LMP1

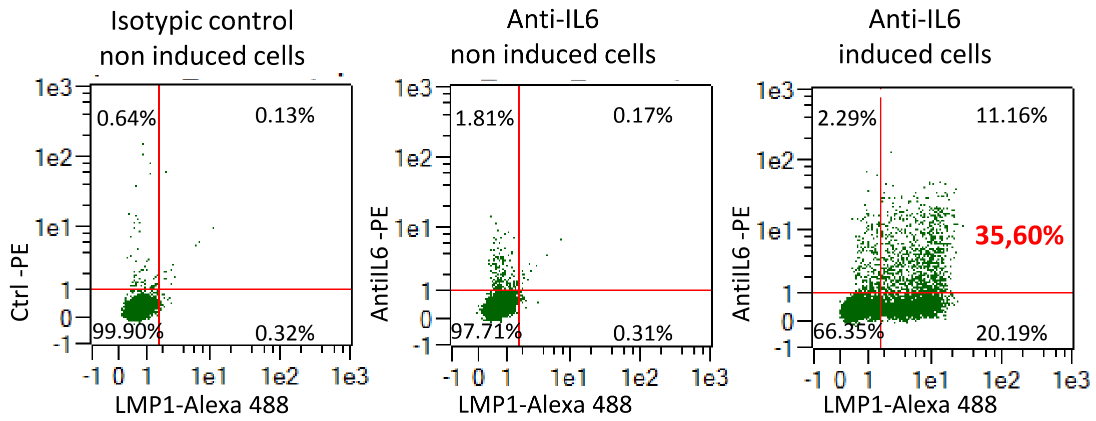

KMH2-del69-LMP1

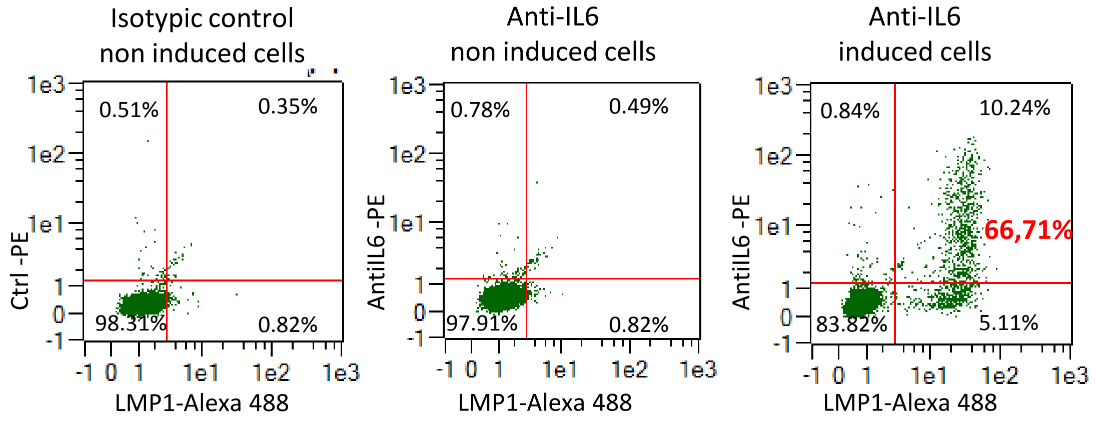

RANTES

KMH2-WT-LMP1

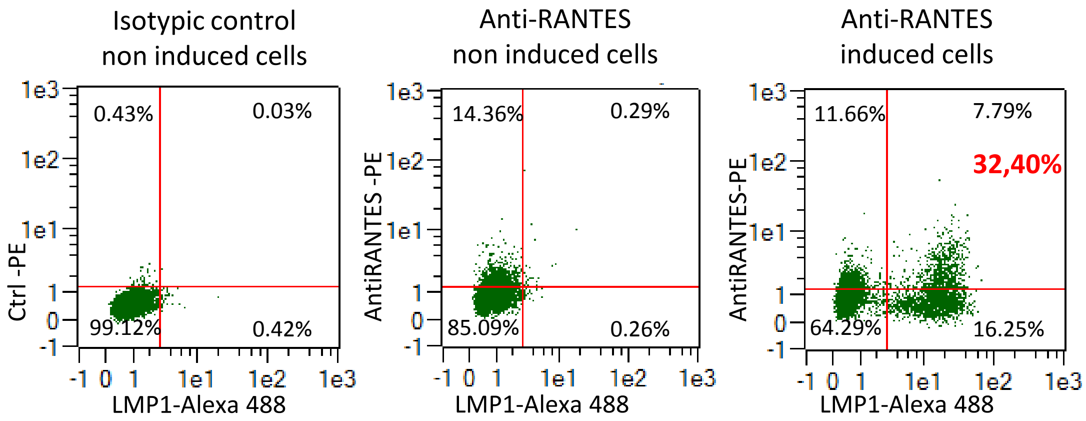

KMH2-del30-LMP1

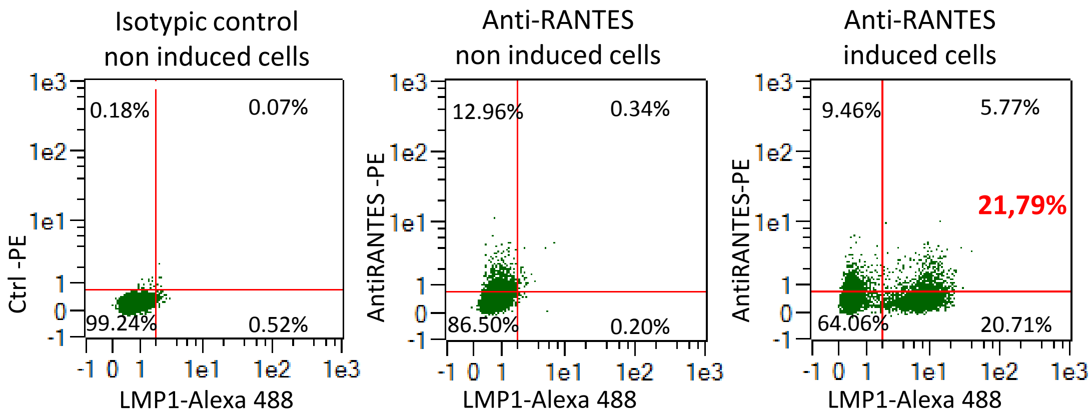

KMH2-del69-LMP1

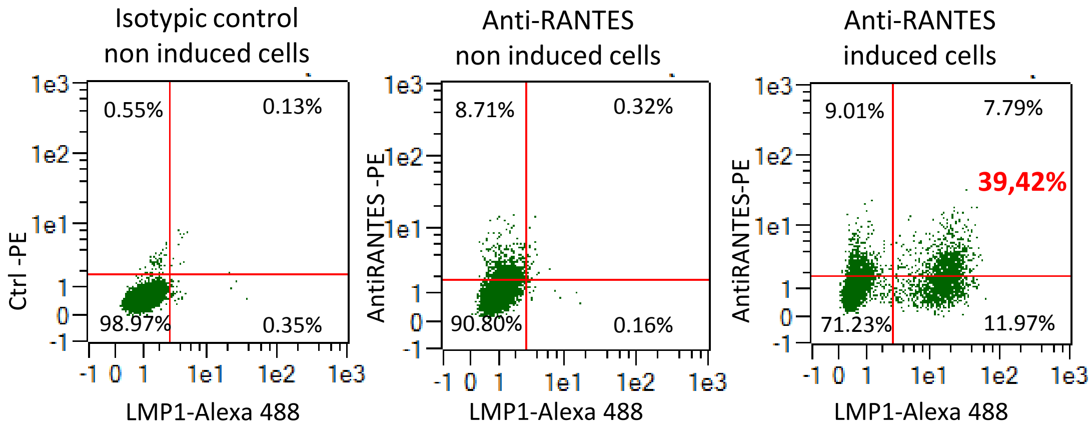

Supplement: Additional file 1 — Cytokine expression by KMH2-pRT-LMP1 expressing cells. Cytometric data plots are presented for each cytokine expressed by the three KMH2-pRT-LMP1 cell lines, with or without induction of LMP1 expression, compared to an isotypic control antibody. X-axis shows LMP1 expression while Y-axis represents cytokine expression. Percentages of cells in each quadrant are given in black. LMP1-positive cells (right upper and lower quadrants) are selectively gated and considered 100% in order to calculate the percentage of LMP1-positive cells expressing the cytokine of interest, indicated in red. Experiments have been conducted in triplicates and pictures shown here represent only one out of three experiments. [file 1743-422X-11-94-S1.pdf]
